# Frequent Epigenetic Inactivation of DIRAS-1 and DIRAS-2 Contributes to Chemo-Resistance in Gliomas

**DOI:** 10.3390/cancers13205113

**Published:** 2021-10-12

**Authors:** Tanja Rothhammer-Hampl, Franziska Liesenberg, Natalie Hansen, Sabine Hoja, Sabit Delic, Guido Reifenberger, Markus J. Riemenschneider

**Affiliations:** 1 Department of Neuropathology, Regensburg University Hospital, 93053 Regensburg, Germany; tanja.rothhammer-hampl@ukr.de (T.R.-H.); sabine.hoja@psk.uni-regensburg.de (S.H.); sabit.delic@mll.com (S.D.); 2 Institute of Neuropathology, Medical Faculty, University Hospital Düsseldorf, Heinrich Heine University, 40225 Düsseldorf, Germany; franziska.liesenberg@googlemail.com (F.L.); nataliehansen@gmx.de (N.H.); reifenberger@med.uni-duesseldorf.de (G.R.); 3 German Cancer Consortium (DKTK), Partner Site Essen/Düsseldorf, 40225 Düsseldorf, Germany

**Keywords:** glioblastoma, p53, chromatin, methylation, histone modification, lomustine

## Abstract

**Simple Summary:**

We investigated the genes *DIRAS-1* and *DIRAS-2* in terms of their regulation and functional relevance in brain tumors (gliomas). We found that in a majority of patients the expression of both genes is strongly downregulated on the mRNA level when comparing tumors with healthy brain tissue. We could show that epigenetic mechanisms account for this downregulation. Both promoter methylation and histone modifications are accountable. We performed experiments in tumor tissues (direct bisulfite sequencing and chromatin-immunoprecipitation) and we treated glioblastoma cell lines in a way to overcome epigenetic inactivation of both genes. When genes were re-expressed, the tumor cells turned out more sensitive to alkylating chemotherapeutic agents such as Lomustin. Changes in intracellular pathways related to p53-mediated DNA damage response may explain for this observation.

**Abstract:**

We previously reported that DIRAS-3 is frequently inactivated in oligodendrogliomas due to promoter hypermethylation and loss of the chromosomal arm 1p. DIRAS-3 inactivation was associated with better overall survival. Consequently, we now investigated regulation and function of its family members DIRAS-1 and DIRAS-2. We found that DIRAS-1 was strongly downregulated in 65% and DIRAS-2 in 100% of analyzed glioma samples compared to non-neoplastic brain tissue (NNB). Moreover, a significant down-regulation of DIRAS-1 and -2 was detected in glioma data obtained from the TCGA database. Mutational analyses did not reveal any inactivating mutations in the *DIRAS-1* and *-2* coding regions. Analysis of the *DIRAS-1* and *-2* promoter methylation status showed significantly higher methylation in *IDH*-mutant astrocytic and *IDH*-mutant and 1p/19q-codeleted oligodendroglial tumors compared to NNB. Treatment of U251MG and Hs683 glioblastoma cells lines with 5-azacytidine led to significant re-expression of DIRAS-1 and -2. For *IDH*-wild-type primary gliomas, however, we did not observe significantly elevated *DIRAS-1* and *-2* promoter methylation levels, but still detected strong downregulation of both DIRAS family members. Additional analyses revealed that DIRAS-1 and -2 expression was also regulated by histone modifications. We observed a shift towards promoter heterochromatinization for *DIRAS-1* and less promoter euchromatinization for *DIRAS-2* in *IDH*-wild-type glioblastomas compared to controls. Treatment of the two glioblastoma cell lines with a histone deacetylase inhibitor led to significant re-expression of DIRAS-1 and -2. Functionally, overexpression of DIRAS-1 and -2 in glioblastoma cells translated into significantly higher sensitivity to lomustine treatment. Analyses of DNA damage markers revealed that DIRAS-1 and -2 may play a role in p53-dependent response to alkylating chemotherapy.

## 1. Introduction

DIRAS-1 and DIRAS-2 are members of the distinct subfamily of small Ras GTPases and were first described by Kontani et al. [1]. The *DIRAS-1* gene is also known as Rig (Ras-related inhibitor of cell growth) and is located on chromosome band 19p13.3, whereas the *DIRAS-2* gene is located on chromosome band 9q22.2 [1,2]. DIRAS-1, in contrast to common oncogenic small GTPases like Ras or Rho family members [3], has been reported as a potential tumor suppressor in human glioblastoma [2], colorectal cancer [4], renal cell carcinoma [5], and ovarian cancer [6], and reduced expression of DIRAS-1 predicts poor prognosis in esophageal squamous cell carcinoma [7]. Bergom and colleagues more closely analyzed the tumor suppressive mechanisms of DIRAS-1 and showed that DIRAS-1 binds to the noncanonical guanine nucleotide exchange factor SmgGDS (Rap1 GTPase-GDP dissociation stimulator 1 = RAP1GDS1) and acts similarly to a dominant-negative small GTPase [3]. SmgGDS showed a stronger binding affinity for DIRAS-1 than for other small GTPases and DIRAS-1, therefore prevented binding of SmgGDS to pro-oncogenic GTPases, such as K-Ras4B, RhoA, and Rap1A. Since DIRAS-1 expression is frequently reduced or lost in malignant tissues, more SmgGDS is available to interact with and activate pro-oncogenic GTPases [3]. Downregulation of DIRAS-1 expression was reported to be partly due to aberrant promoter methylation in esophageal squamous cell carcinoma, renal cell carcinoma, and colorectal cancer [4,5,7] and increased DIRAS-1 expression was shown in one renal cell carcinoma cell line after treatment with a histone deacetylase inhibitor [8]. 

The role of DIRAS-2 in human disease was first studied in attention deficit/hyperactivity disorders (ADHD) and co-morbid impulsive disorders, since the chromosomal region 9q22, where the *DIRAS-2* gene is located, showed an association with this type of disease in genome-wide association studies [9]. More recent publications, however, also suggested a role of DIRAS-2 in malignant transformation. Sutton and colleagues showed that expression of DIRAS-1 and DIRAS-2 is downregulated in ovarian cancer and associated with decreased disease-free and overall survival [6]. DIRAS-1 or -2 induced cell death in murine ovarian cancer cells via autophagy and the authors suggested that both DIRAS isoforms might also play a role in human autophagy [6]. A study published by Ogita et al. showed that DIRAS-2, analogous to DIRAS-1, forms a high affinity complex with SmgGDS, which then might prevent interaction of SmgGDS with other pro-oncogenic small GTPases and, therefore, also suggested a potential tumor suppressive role for DIRAS-2 [10]. In clear cell renal cell carcinoma (ccRCC), however, DIRAS-2 exhibited a potential oncogenic function, which is in contrast to all other published studies [11]. The authors reported an upregulation of *DIRAS-2* mRNA in ccRCC compared to normal kidney tissue by analyzing TCGA and Oncomine databases and they uncovered a specific function of DIRAS-2 in ccRCC as an activator of MAPK signaling pathway in the absence of the von Hippel–Lindau protein (pVHL) [11].

The cited studies provide hints on a relevance of DIRAS-1 and DIRAS-2 alterations in various types of cancer. However, regulatory mechanisms have as yet been incompletely understood and functional conclusions are, in part, contradictory between cancer entities. We here investigated the exact mechanisms of DIRAS-1 and -2 regulation in gliomas and its functional implications in terms of cell proliferation and sensitivity to DNA alkylating chemotherapy.

## 2. Materials and Methods

### 2.1. Tumor Samples, Cell Lines

Glioma tissue samples were retrieved from the archives of the Institute of Neuropathology, Heinrich Heine University, Düsseldorf, Germany, and investigated as approved by the institutional review board (study number 2830). Tumors were classified according to criteria of the World Health Organization (WHO) classification of tumors of the central nervous system 2016 [12]. Only samples with a tumor cell content of 80% or more were selected for investigation. The tumor cohort comprised 34 human gliomas, including 12 glioblastomas, *IDH*-wild-type, WHO grade IV (GBIV); three anaplastic astrocytomas, *IDH*-wild-type, WHO grade III (AAIII); one diffuse astrocytoma, *IDH*-wild-type, WHO grade II (AII); four anaplastic astrocytomas, *IDH*-mutant, WHO grade III (AAIII); four diffuse astrocytomas, *IDH*-mutant, WHO grade II (AII); eight anaplastic oligodendrogliomas, *IDH*-mutant and 1p/19-codeleted, WHO grade III (OIII); two oligodendrogliomas, *IDH*-mutant and 1p/19-codeleted, WHO grade II (OII). Four non-neoplastic brain samples from different individuals (NB1–NB4) were used as reference. As a positive control for methylation studies, commercially available hypermethylated DNA was employed (Cat. No. S7821; Millipore, USA). U251MG and Hs683 glioblastoma cells were obtained from Cell Lines Service GmbH (Eppelheim, Germany).

### 2.2. In Silico Analysis of Glioblastoma Samples from the Cancer Genome Atlas Database

Prenormalized expression data sets from glioblastoma samples were obtained from the cancer genome atlas data portal (URL: https://tcga-data.nci.nih.gov, 2 December 2014) [13] and analyzed as previously published by Schulze et al. [14]. For analysis of *DIRAS-1* and *DIRAS-2* coding mutations the glioblastoma sample data set published by Brennan et al. [13] was assessed by using the cBioPortal webpage (https://www.cbioportal.org/, 22 June 2021) [15,16].

### 2.3. Real-Time Reverse Transcription PCR Analysis

Total RNA was reverse transcribed and DIRAS-1 and -2 mRNA expression was determined by real-time PCR using the StepOnePlusTM detection system (Applied Biosystems brand of Thermofisher Scientific, Waltham, MA, USA), by continuous measurement of PCR product amount by incorporation of SybrGreen fluorescent dye. Transcript levels of either ARF1 (ADP-ribosylation factor 1) or GAPDH (glyceraldehyde-3phosphate dehydrogenase) were used for normalization. The target gene/ARF1 or GAPDH mRNA ratios in individual gliomas were calculated relative to the mean target gene/ARF1 or GAPDH mRNA ratio in commercially available RNA from non-neoplastic brain tissue (human normal brain, human fetal brain, Takara Bio Europe, France; human adult brain, Stratagene, La Jolla, CA, USA) (Primer sequences are listed in Table S2).

### 2.4. Mutational Analyses

Single-strand conformation polymorphism (SSCP)/heteroduplex analysis was performed to screen for mutations in the *DIRAS-1* and *DIRAS-2* coding sequences. PCR products were separated by electrophoresis on 10%–12% non-denaturing polyacrylamide gels at room temperature and at 4° C. After electrophoresis, the SSCP/heteroduplex band patterns were visualized by silver staining of the gels. In case of aberrant band patterns, PCR products were sequenced (Primer sequences are listed in Table S2). Detected mutations were somatic silent (G was replaced by C; nucleotide triplet code: GGG > GGC). The replacement of G by C did not result in altered protein sequence as both nucleotide triplets code for the amino acid glycine.

### 2.5. IDH1 and IDH2 Mutation Analyses and Evaluation for 1p/19q Loss of Heterozygosity

*IDH1* (codon 131 and 132) and *IDH2* (codon 172) mutations were determined by PCR-amplification, followed by direct Sanger sequencing using the BigDye Terminator v1.1 Cycle Sequencing Kit (Thermo Fisher Scientific) on the SeqStudio Genetic Analyzer (Thermo Fisher Scientific) and 1p/19q loss of heterozygosity was determined by PCR-based microsatellite analysis of five markers on chromosomal arm 1p and three markers on 19q according to quality controlled protocols established in our laboratory (Primer sequences are listed in Table S2) [17].

### 2.6. DNA Methylation Analysis Using Sodium Bisulfite Sequencing 

Sodium bisulfite treatment of DNA was performed using the EpiTect Bisulfite Kit (Cat No. 59104, Qiagen, Hilden, Germany). For *DIRAS-1* methylation status, a fragment from CpG island 114 (hg38), covering the first 7 CpG sites located 303-bases 5’ to the transcription start site, was PCR-amplified. For *DIRAS-2*, a fragment covering 14 CpG sites of CpG island 20 (hg38) located in exon 1 (13 CpG sites) and intron 1 (1 CpG site) was amplified. Methylation status of the CpG sites was determined by direct sequencing of PCR product and methylation was rated using the following scale: 0, completely unmethylated; 1, weak methylated signal; 2, methylated signal approximately equal to unmethylated signal; 3, methylated signal markedly stronger than unmethylated signal. Based on this rating, a cumulative promoter methylation score was calculated for each tumor. Statistical significance was analyzed by employing One-way ANOVA followed by Dunnett’s multiple comparison for significance using GraphPad Prism Version 7 (GraphPad Software Inc., San Diego, CA, USA). (Primer sequences are listed in Table S2). 

### 2.7. 5-Aza-2’-Deoxycytidine (AZA) and Trichostatin A (TSA) Treatment of Glioblastoma Cell Lines

Two *IDH*-wild-type glioblastoma cell lines (U251MG and Hs683) were either grown with or without the demethylating agent AZA (1 µM for 72 h, AZA), the histone deacetylase inhibitor TSA (1 µM for 48 h) or a combination of both agents. After harvesting the cells and extracting the mRNA, expression of DIRAS-1 and DIRAS-2 transcripts of AZA, TSA, or Aza plus TSA treated cells as well as untreated control cells was assessed by real-time reverse transcription PCR analysis (Primer sequences are listed in Table S2). Results were reproduced in at least three independent biological experiments. Statistical significance was analyzed by unpaired student’s *t*-test using GraphPad Prism Version 7.

### 2.8. Chromatin Immunoprecipitation (ChIP) Assays

ChIP was performed from TSA-treated glioblastoma cells as well as from fresh frozen glioblastoma (all *IDH*-wild-type) tissues, as described by Jiang et al [18]. Briefly, DNA and proteins were cross-linked with 1% formaldehyde and nuclei were isolated, purified, and then resuspended in SDS-lysis buffer. Genomic DNA was sheared to 200–800-bp fragments by sonication (Covaris, USA). After shearing, samples were pre-cleared with protein A agarose/salmon sperm DNA to reduce nonspecific binding. A 5% sample volume was saved as input control. The rest of the lysate was used for immunoprecipitation at 4 °C overnight with acetylated histone H3 antibody (associated with euchromatin) or the histone H3 trimethylated at lysine residue 9 antibody (H3K9me3, associated with heterochromatin). Rabbit anti-human IgG fraction served as a negative isotype control (all antibodies from Upstate brand of Fisher scientific, part of Thermofisher Scientific, Waltham, MA, USA). Antibody/histone/DNA complexes were then collected and the histone/DNA complexes eluted from the antibodies. After reversing the histone–DNA cross-link, the DNA was retrieved by proteinase K digestion and subsequent phenol/chloroform extraction. Immunoprecipitated DNA was assessed by real-time PCR analysis targeting the promoter sequences of *DIRAS-1* and *DIRAS-2* and normalized to the respective input fraction as a reference. *GAPDH* was used as a negative control gene associated with euchromatin and not regulated by histone modifications [19]. Results obtained from glioblastoma cells were reproduced in at least three independent biological experiments and statistical significance was analyzed by unpaired student’s *t*-test using GraphPad Prism Version 7. For fresh frozen glioblastoma tissues, the results are based on the measurement of three technical replicates caused by the restricted amount of immunoprecipitated DNA (Primer sequences are listed in Table S2).

### 2.9. Cell Proliferation Assay

Glioblastoma cells were seeded in triplicates (96-well plates, 3000 cells in 100 μL per well) and transfected with DIRAS-1, DIRAS-2, or control plasmids, respectively. Forty-eight hours after transfection a chemiluminescent BrdU Cell Proliferation ELISA (Roche Diagnostics GmbH, Germany) was performed according to the manufacturer’s instructions (BrdU incorporation for 4 h). Results were reproduced in at least three independent biological experiments and statistical significance was analyzed by ordinary one-way ANOVA (analysis of variance) followed by Holm–Sidak’s multiple comparison test using GraphPad Prism Version 7.

### 2.10. Temozolomide and Lomustine Chemosensitivity Assay

Cells were seeded in triplicates (96-well plates, 500 cells in 100 µL per well). Then, 24 h later, cells were transfected with either the control plasmid or DIRAS-1 and DIRAS-2 expression plasmids, respectively. Twenty-four h after the transfection, various concentrations of temozolomide (1–4000 µM) or lomustine (4–250 µM) were added, respectively. Cell viability was measured 72 h later by detecting relative fluorescent units using the resazurin reagent (R&D Systems, Minneapolis, MN, USA). The half maximal inhibitory concentration (IC50) value was determined as the concentration resulting in a 50% growth reduction compared to control cell growth (i.e., cells that did not obtain temozolomide or lomustine). To perform the curve fitting, chemotherapeutic concentrations were log-transformed, means of fluorescent units of each chemotherapeutic concentration were normalized (0%: smallest mean in each data set; 100%: largest mean in each data set) and then fitted using a sigmoidal dose response (variable slope) fitting model (4-parameter logistics) with the fitting method of least squares (ordinary) fit (GraphPad Prism Version 7). IC50 values of control transfected and either Diras-1- or Diras-2-transfected cells of at least three independent experiments were compared and statistical significance was analyzed by repeated measures of one-way ANOVA (analysis of variance) followed by Dunnett’s multiple comparisons test using GraphPad prism version 7. Absolute IC50 concentration for temozolomide was in the range of 1357 ± 34 µM for U251 and 442 ± 256.1 µM for Hs683, and IC50 for lomustine was in the range of 118.5 ± 82.7 µM for U251 and 47.0 ± 19.3 µM for Hs683 (control transfected cells).

### 2.11. Protein Expression Analysis by Western Blotting

For analysis of DNA-damage markers, cells were transfected with either DIRAS-1, DIRAS-2, or control plasmids, and, 48 h after transfection, cells were treated with 0, 25 µM, or 50 µM lomustine, respectively, for 24 h. For analysis of DIRAS-1 and -2 overexpression, cells were harvested 48 h after transfection and total protein was extracted. For protein isolation, 10^6^ cells were washed in 1× PBS and lysed in 100 µL RIPA buffer. The protein concentration was determined using the DC protein assay according to the manufacturer’s protocol (Bio-Rad Laboratories Inc., Hercules, CA, USA). Balanced amounts of cell proteins (20 µg for DIRAS-1 and -2 analysis, 40 µg for DNA-damage marker analysis) were denatured at 95 °C for 5 min after addition of Laemmli sample buffer containing β-mercaptoethanol and subsequently separated by polyacrylamide gel electrophoresis using Mini-PROTEAN TGX™ Precast Gels (4–20%, Bio-Rad Laboratories Inc., Hercules, CA, USA). After transferring the proteins onto nitrocellulose membranes (Bio-Rad Laboratories Inc., Hercules, CA, USA), the membranes were blocked in 5% bovine serum albumin/TBS for 1 h and incubated with a 1:1000 dilution of primary antibodies (DNA damage antibody sampler kit: phospho-ATM, phospho-ATR, phospho-BRCA1, phospho-Chk1, phospho-Chk2, phospho-p53, phospho-H2AX, Cell Signaling Technology Inc., USA; DIRAS-1 #12634-1-AP, DIRAS-2 #15557-1-AP, Proteintech, Europe, Manchester, UK), respectively, overnight at 4 °C. For the tubulin loading control, membranes were blocked in 5% BSA/TBS for 1 h and incubated with a 1:3333 dilution of anti-α-tubulin antibody (Sigma Aldrich, St. Louis, MO, USA). A 1:5000 dilution of anti-rabbit-HRP or anti-mouse-HRP was used as secondary antibody (anti-rabbit: Thermo Fisher Scientific, Waltham, USA, anti-mouse: Santa Cruz Biotechnology Inc., Dallas, TX, USA). Bands were then detected using the SuperSignal West Pico chemiluminescence substrate (ThermoFisher Scientific, Watlham, MA, USA) and the ImageQuant LAS 4000 CCD camera system and quantified using the ImageQuant TL software version 8.2.0.0 (GE Healthcare Lifesciences, Boston, MA, USA). 

## 3. Results

### 3.1. DIRAS-1 and DIRAS-2 Are Transcriptionally Downregulated in the Majority of the Investigated Gliomas and in the TCGA Data Set

**Figure 1:**
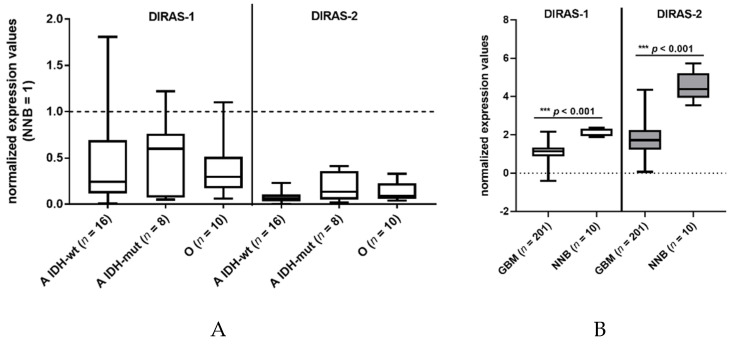
DIRAS-1 and DIRAS-2 expression in gliomas and non-neoplastic brain tissues. (**A**) Analysis of DIRAS-1 and DIRAS-2 mRNA expression in 34 glioma samples (A: astrocytic and O: oligodendroglial tumors, *IDH*-wt: Wild-type *IDH* status, *IDH*-mut: mutated *IDH* status) (**B**) Analysis of DIRAS-1 and DIRAS-2 mRNA expression in data obtained from The Cancer Genome Atlas database (GBM: glioblastoma, NNB: non-neoplastic brain) [13].) Original figure in Figure S1.

We investigated the expression of DIRAS-1 transcripts and detected decreased mRNA levels (<0.5-fold relative to non-neoplastic brain tissue) in 22 out of 34 gliomas (65%) and intermediate mRNA levels (between 0.5 and <1 relative to non-neoplastic brain tissue) in eight out of 34 gliomas (24%) (Table S1). This finding confirms data already reported by Ellis et al [2]. DIRAS-2 mRNA expression was analyzed in the same 34 glioma patients. All analyzed glioma tissues (100%) showed decreased mRNA levels (< 0.5-fold) compared to non-neoplastic brain tissue (Figure 1A, Table S1). Additional analysis of data obtained in a larger cohort from The Cancer Genome Atlas database [13] also showed a significant downregulation of DIRAS-1 and -2 transcripts in glioblastoma samples compared to non-neoplastic brain samples (Figure 1B).

### 3.2. Lack of Inactivating Mutations in DIRAS-1 and DIRAS-2 Coding Sequence

Mutational analysis of the entire coding region of *DIRAS-1* detected aberrant band pattern in seven of 30 gliomas for *DIRAS-1*. Subsequent sequencing of the PCR products showed somatic silent mutations in five of seven gliomas. For two of seven gliomas showing aberrant band pattern, sequencing results could not be obtained. Mutational analysis of the entire coding region of *DIRAS-2* did not detect somatic mutations in any of the 32 gliomas analyzed. Additional analysis of a larger glioblastoma data set (291 samples) published by Brennan et al. [13] did also not reveal any coding mutations in the *DIRAS-1* or *DIRAS-2* gene.

### 3.3. Promoter Hypermethylation Partially Accounts for the Downregulation of DIRAS-1 and -2 Expression

**Figure 2:**
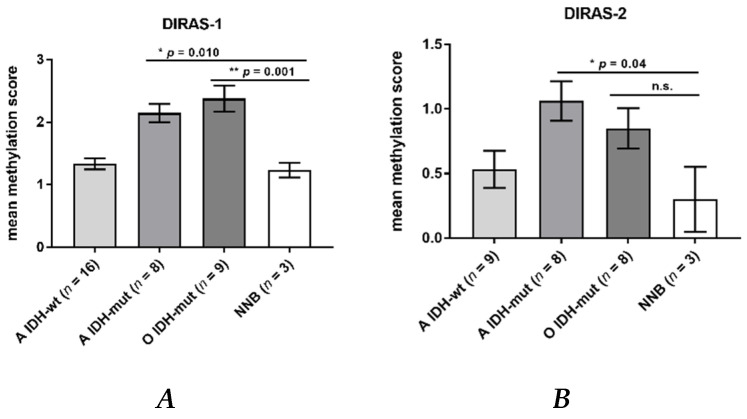
*DIRAS-1* and *DIRAS*-2 CpG methylation status in gliomas and non-neoplastic brain tissues. (**A**) *DIRAS-1* CpG island methylation scores (A: astrocytic and O: oligodendroglial tumors, *IDH*-wt: Wild-type *IDH* status, *IDH*-mut: mutated *IDH* status) and three non-neoplastic brain tissues. (**B**) *DIRAS-2* CpG island methylation scores for 25 glioma tissues and three non-neoplastic brain tissues (n.s.: not significant). Original figure in Figure S1.

**Figure 3:**
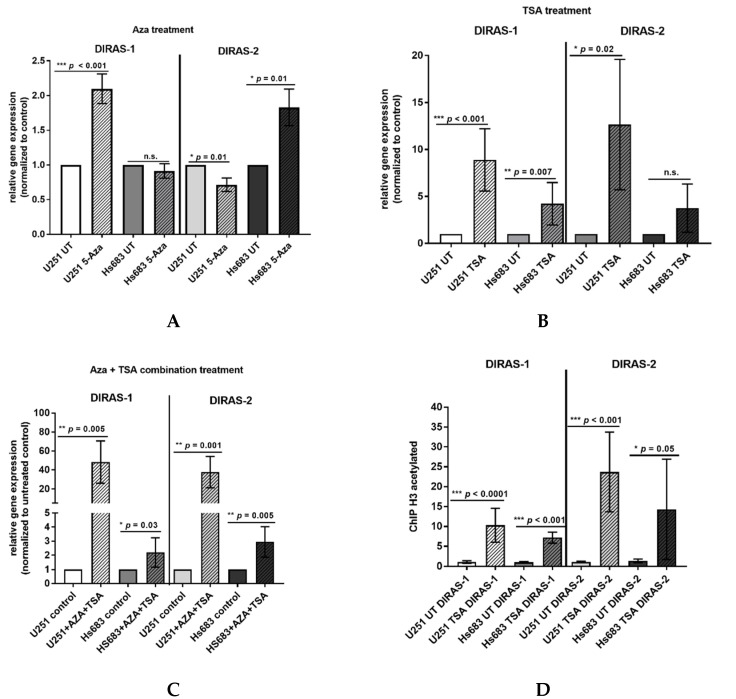
Hypermethylation and histone modifications account for transcriptional downregulation of DIRAS-1 and DIRAS-2. (**A**) Increase in DIRAS-1 and DIRAS-2 mRNA expression in U251MG and Hs683 cells after treatment with 5-azacytidine, (**B**) after treatment with trichostatin A (TSA), and (**C**) after combinational treatment with 5-azacytidine and trichostatin A. (**D**) Increase in *DIRAS-1* and *DIRAS-2* promoter DNA bound to acetylated H3, indicating *DIRAS-1* and *DIRAS-2* promoter euchromatinization after TSA treatment in U251MG and Hs683 cells (n.s.: not significant, * *p* ≤ 0.05, ** *p* ≤ 0.002, *** *p* ≤ 0.001). Original figure in Figure S1.

Direct bisulfite sequencing showed significant hypermethylation of the analyzed region of the *DIRAS-1* gene (CpG island 114) in *IDH*-mutant astrocytic tumors (mean methylation score: 2.15, *p* = 0.01) and in *IDH*-mutant and 1p/19q-codeleted oligodendroglial tumors (mean methylation score of 2.38, *p* = 0.001) compared to non-neoplastic brain tissue (mean methylation score: 1.23). The mean methylation score of astrocytic tumors with *IDH*-wild-type status (mean methylation score: 1.33, *p* = n.s.) did not significantly differ from non-neoplastic brain tissue (Figure 2A, Table S1). The mean methylation score of the two analyzed glioblastoma cell lines was comparable to the methylation score of hypermethylated tumors (mean methylation score cell lines: 2.43, Table S1). Analysis of CpG island 20 of the *DIRAS-2* gene showed significantly different methylation between non-neoplastic brain tissue and *IDH*-mutant astrocytic tumors (mean methylation score: 1.08, *p* = 0.04). The mean methylation scores of *IDH*-wild-type astrocytic tumors (mean methylation score: 0.53, *p* = not significant) and of *IDH*-mutant and 1p/19q-codeleted oligodendroglial tumors (mean methylation score: 0.85, *p* = not significant) were not significantly different from non-neoplastic brain tissue (mean methylation score: 0.3). However, the absolute degree of methylation of CpG island 20 of the *DIRAS-2* gene was quite low in all samples analyzed (Figure 2B, Table S1). The mean methylation score of the two analyzed glioblastoma cell lines was again comparable to the stronger methylated glioma tissues (mean methylation score cell lines: 1.2, Table S1). Treatment of the glioblastoma cell lines U251MG and Hs683 with AZA induced a significant increase of DIRAS-1 transcripts in U251MG cells (2.07-fold to control, *p* < 0.001) and of DIRAS-2 transcripts in Hs683 cells (1.79-fold to control, *p* = 0.01). However, DIRAS-1 expression in Hs683 cells and DIRAS-2 expression in U251MG cells were not significantly altered by AZA treatment (Figure 3A). Altogether, these data show that aberrant CpG island methylation may contribute to the decreased DIRAS-1 and DIRAS-2 mRNA levels in selected gliomas but likely is not the only factor. Thus, we subsequently analyzed whether histone modifications may also contribute to DIRAS-1 and -2 downregulation in gliomas. 

### 3.4. In Vitro Treatment of Glioblastoma Cell Lines with TSA Shows a Role of Histone Modifications in DIRAS-1 and DIRAS-2 Downregulation

The two glioblastoma cell lines, U251MG and Hs683, were treated with TSA to analyze the role of histone modifications in DIRAS-1 and -2 transcriptional regulation. In vitro treatment with TSA causes increased acetylation of histones and, therefore, leads to transcriptionally active euchromatin. The TSA-treated cells showed a significant increase in DIRAS-1 and DIRAS-2 mRNA expression in U251MG cells (DIRAS-1: 8.89-fold, *p* < 0.001; DIRAS-2: 12.65-fold, *p* = 0.02) and a significant increase of DIRAS-1 expression in Hs683 cells (4.21-fold, *p* = 0.007) compared to controls. An increase of DIRAS-2 expression in Hs683 cells after TSA treatment was also observed (DIRAS-2: 3.76-fold, *p* = 0.0757); however, this increase was not significant (Figure 3B). The combined treatment of U251MG cells with AZA and TSA strongly induced DIRAS-1 and DIRAS-2 mRNA expression (DIRAS-1: 48.3-fold, *p* = 0.005, DIRAS-2: 37.6-fold, *p* = 0.001) and suggests synergistic effects of histone modifications and promoter methylation. In Hs683 cells we also observed a significant increase of DIRAS-1 and -2 with combinatory treatment; however, there was no synergistic effect, as the x-fold increase was comparable to TSA treatment alone (DIRAS-1: 2.20-fold, *p* = 0.03, DIRAS-2: 2.95-fold, *p* = 0.005) (Figure 3C). We next analyzed U251MG and Hs683 cell lines for a potential promoter euchromatinization and found a significant increase of the DIRAS-1 and DIRAS-2 promoter DNA bound to acetylated H3 in both cell lines after TSA treatment compared to control (U251MG: DIRAS-1: 10.3-fold, *p* < 0.001; DIRAS-2: 23.7-fold, *p* < 0.001; Hs683: DIRAS-1: 7.2-fold, *p* < 0.001; DIRAS-2: 14.3-fold, *p* = 0.05; Figure 3D).

We next analyzed U251MG and Hs683 cell lines for a potential promoter euchro-matinization and found a significant increase of the *DIRAS-1* and *DIRAS-2* promoter DNA bound to acetylated H3 in both cell lines after TSA treatment compared to control (U251MG: *DIRAS-1*: 10.3-fold, *p* < 0.001; *DIRAS-2:* 23.7-fold, *p* < 0.001; Hs683: *DIRAS-1*: 7.2-fold, *p* < 0.001; *DIRAS-2*: 14.3-fold, *p* = 0.05; Figure 3D).

### 3.5. ChIP Analysis of Primary Glioblastoma Tissues Indicates Heterochromatinization of the DIRAS-1 and -2 Promoter

**Figure 4:**
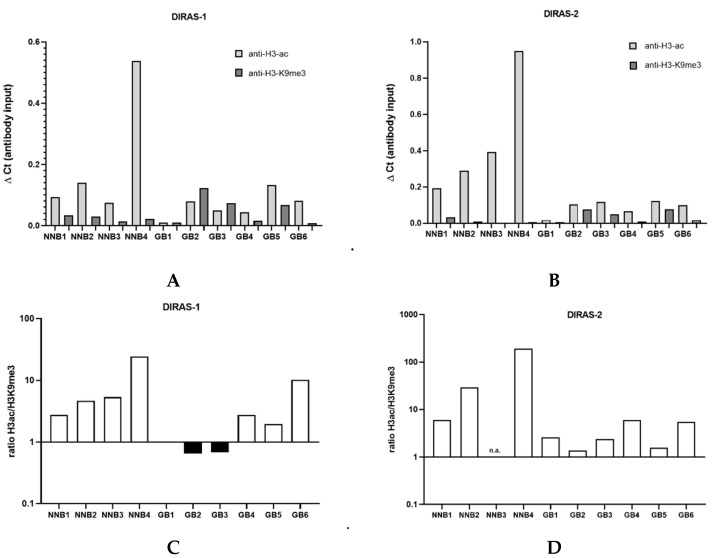
Chromatin immunoprecipitation analysis of glioblastomas and non-neoplastic brain tissues. (**A**) ChIP analysis for *DIRAS-1* using anti-H3-ac and anti-H3-K9me3. (**B**) ChIP analysis for *DIRAS-2*. (**C**) Ratio between H3ac- and H3K9me3-bound *DIRAS-1* DNA. (**D**) Ratio for *DIRAS-2* DNA. Original figure in Figure S1.

We next prepared the chromatin of six *IDH*-wild-type glioblastoma tissues as well as four non-neoplastic brain tissue samples. Chromatin immunoprecipitation analysis showed that in the four non-neoplastic brain tissues the ratio between H3ac- and H3K9me3-bound DNA strongly exceeded 1 (*DIRAS-1*: non-neoplastic brain=NNB1: ratio 2.8, NNB 2: ratio 4.8, NNB3: ratio 5.4, NNB4: ratio 24.5; *DIRAS-2*: NNB1: ratio 6.1, NNB2: 29.0, NNB3: ratio not determined due to lack of H3K9me3-bound DNA, NNB4: 189.8), indicating a prevalent euchromatic stage of the H3-bound *DIRAS-1* and *DIRAS-2* promoter (Figure 4C,D). In glioblastoma tissues, in contrast, three out of the six investigated glioblastomas (GB1, GB2, and GB3) exhibited a prevailing heterochromatinization of the H3-bound *DIRAS-1* promoter (ratio H3ac/H3K9me3 ≤ 1; Figure 4A,C). For *DIRAS-2* we did not observe H3ac/H3K9me3 ratios ≤ 1 in glioblastoma tissues; however, the H3ac/H3K9 ratio in all tumors (as for DIRAS-1) was markedly lower than in non-neoplastic brain tissues (range ratio NNB tissue: 6.1- 189.8, range ratio GBM tissue: 1.4–3.2, Figure 4B,D). Altogether, these data indicate that promoter heterochromatinization in gliomas relevantly contributes to DIRAS-1 and DIRAS-2 transcriptional downregulation.

### 3.6. Functional Analyses of the Role of DIRAS-1 and DIRAS-2 in U251MG and Hs683 Glioblastoma Cells

**Figure 5:**
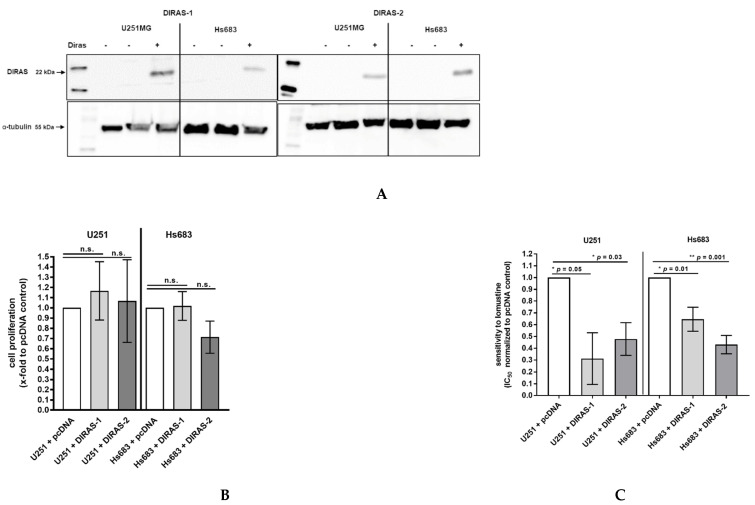
Functional analyses of the role of DIRAS-1 and DIRAS-2 in human gliomas. (**A**) Western blot analysis showing DIRAS-1 and DIRAS-2 overexpression in U251MG and Hs683 cells. Detailed information about Western Blots can be found in the supplementary materials. (**B**) Cell proliferation after overexpression of DIRAS-1 or DIRAS-2 and (**C**) chemosensitivity to lomustin after overexpression of DIRAS-1 or DIRAS-2 in U251MG and Hs683 cells (n.s.: not significant, * *p* ≤ 0.05, ** *p* ≤ 0.002). Original figure in Figure S1.

To analyze the functional role of DIRAS-1 and DIRAS-2, both proteins were over-expressed in U251MG and Hs683 glioblastoma cells by transient transfection with plasmids coding for DIRAS-1 or DIRAS-2. Expression of DIRAS-1 and DIRAS-2 proteins 48 h after transfection was confirmed by Western blot analysis (Figure 5A). Cell proliferation in DIRAS-1 or -2 overexpressing cells was analyzed by BrdU incorporation ELISA assay. There was no significant difference in BrdU incorporation and, therefore, the cell proliferation rate in DIRAS-1 or -2 transfected was similar to that in control transfected cells (Figure 5B). 

Since there had been reports that DIRAS-3, a third member of the distinct subfamily of small Ras GTPases, enhanced sensitivity of ovarian cancer cells to chemotherapeutic agents [20], we were especially interested if DIRAS-1 or DIRAS-2 overexpression can sensitize glioblastoma cells to chemotherapeutic agents such as temozolomide or nitrosourea (e.g., lomustine = chlorethyl-cyclohexyl-nitroso-urea). We therefore analyzed the half maximal inhibitory concentration (IC50) for temozolomide and lomustine in DIRAS-1 or -2 overexpressing and control cells using the two cell lines U251MG and Hs683. We did not observe significant differences in IC50 values in DIRAS-1 or DIRAS-2 overexpressing cells compared to control transfected cells after treatment with temozolomide. However, we observed significantly lower IC50 values in DIRAS-1 or -2 overexpressing U251MG and Hs683 cells when treated with lomustine, indicating that DIRAS-1 or DIRAS-2 overexpression sensitizes glioblastoma cells to treatment with nitrosourea agents (Figure 5C). 

**Figure 6:**
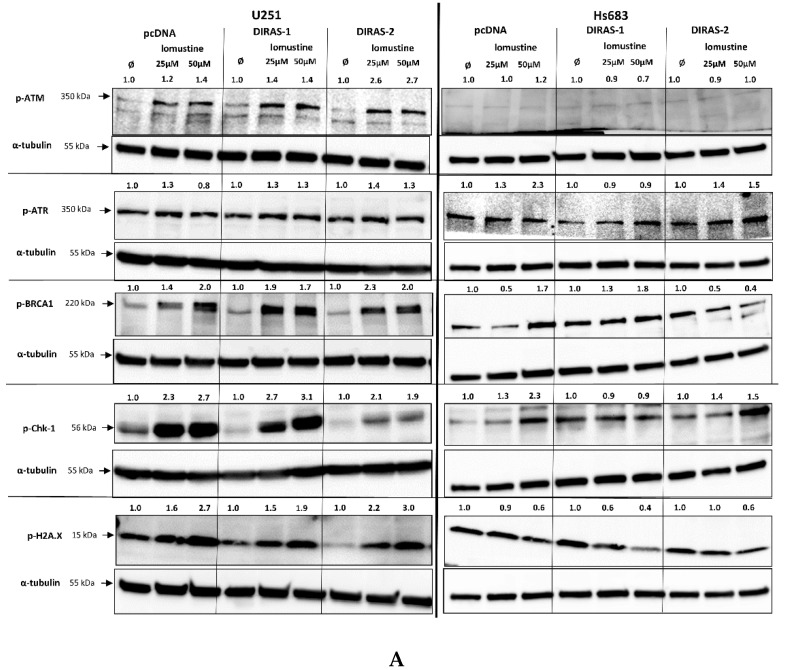

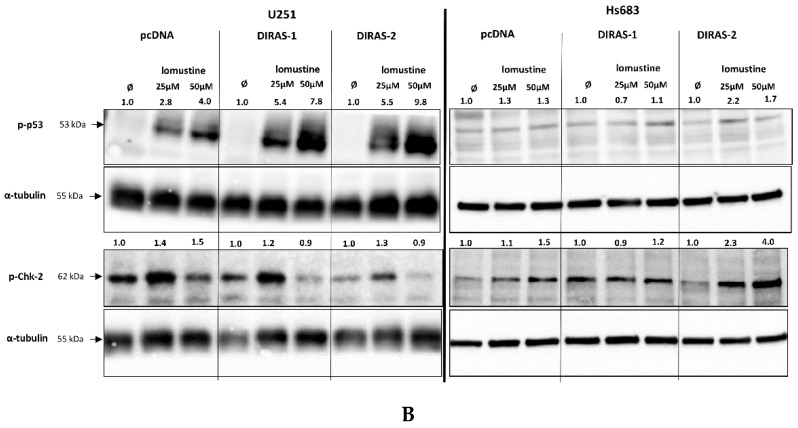
Western blot analyses of phosphorylation of DNA damage markers in DIRAS-1 or DIRAS-2 overexpressing glioblastoma cells after lomustin treatment. (**A**) Phospho-ATM (ataxia-telangiectasia-mutated gene), phospho-ATR (ataxia-telangiectasia and Rad3 related), phospho-BRCA-1 (breast cancer 1 gene), phospho-Chk-1 (checkpoint kinase 1), and phospho-H2A.X (H2A histone family member X), (**B**) phospho-p53 (transformation-related protein 53) and phospho-Chk-2 (checkpoint kinase 2). Original figure in Figure S1. Detailed information about Western Blots can be found in the supplementary materials section.

To further investigate the effect of lomustine on a molecular level, we analyzed the phosphorylation of proteins involved in DNA-damage response. Treatment of U251MG and Hs683 glioblastoma cells with two different lomustine concentrations for 24 h led to increased phosphorylation of several DNA-damage response markers, such as BRCA-1 (breast cancer 1 gene), ATM (ataxia-telangiectasia-mutated gene), ATR (ataxia-telangiectasia and Rad3 related), Chk-1 (checkpoint kinase 1), and H2A.X (H2A histone family member X), but we did not observe differences in phosphorylation between control and DIRAS-1 or -2 transfected cells (Figure 6A). Interestingly, when looking at phosphorylation of p53 (tumor protein 53), we found a strong increase after lomustine treatment in DIRAS-1 and in DIRAS-2 transfected U251MG cells compared to control transfected cells (Figure 6B). Albeit to a lower extent, in Hs683 cells we observed a similar effect in DIRAS-2 transfected cells. Overexpression of DIRAS-1 or DIRAS-2 in U251 and Hs683 cells did not lead to increased total p53 protein expression; therefore, we can exclude that the observed effects were due to transcriptional regulation. Moreover, in DIRAS-2 transfected Hs683 cells compared to controls, there was also a visible increase in Chk-2 (checkpoint kinase 2) phosphorylation, which lies upstream of p53. As such, our data suggest that DIRAS-1 and -2 may be involved in p53-mediated DNA damage response.

## 4. Discussion

Studies on the regulation and function of DIRAS-3 have been published on various types of cancers including glioma [20,21,22,23,24,25,26,27]. DIRAS-3 belongs to the same family of small Ras GTPases as DIRAS-1 and -2. In contrast, regulation and function of DIRAS-1 and -2 in gliomas has not been addressed in detail so far. Reduced or lost expression of DIRAS-1 in astrocytic tumors, especially in glioblastomas, had been reported by Ellis et al. [2], while expression of DIRAS-1 was abundant in two out of three oligodendrogliomas in that study. In our analyses we confirmed the downregulation of DIRAS-1 mRNA expression in *IDH*-mutant astrocytic tumors, but also found DIRAS-1 transcript levels strongly reduced in eight out of 10 *IDH*-mutant and 1p/19q-codeleted oligodendroglioma samples. This discrepancy could be due to the low sample numbers analyzed in the study performed by Ellis and colleagues [2]. For DIRAS-2 we detected a pronounced down-regulation in all glioma samples analyzed, including *IDH*-mutant astrocytic and oligodendroglial tumors as well as *IDH*-wild-type glioblastomas. These findings were confirmed by analyzing a larger data set obtained from The Cancer Genome Atlas database, including 201 glioblastoma tissues and 10 non-neoplastic brain controls [13].

Due to the absence of any inactivating mutations in the *DIRAS-1* and *-2* coding regions, we were interested in whether the loss of expression of both DIRAS family members in tumor samples might be caused by promoter hypermethylation. Direct bisulfite sequencing revealed significantly increased methylation scores of the analyzed CpG island of *DIRAS-1* in *IDH*-mutant astrocytic as well as in *IDH*-mutant and 1p/19q-codeleted oligodendroglial tumors but did not detect significantly increased *DIRAS-1* promoter methylation in *IDH*-wild-type astrocytic tumors. This finding might be due to the fact that *IDH* mutation leads to a CpG island methylator phenotype (CIMP) in gliomas, which causes genome-wide extensive DNA hypermethylation [28], likely including hypermethylation of the CpG island 114 in the *DIRAS-1* promoter region. However, DNA hypermethylation alone may not be sufficient to down-regulate DIRAS-1, as we also observed reduced DIRAS-1 expression in *IDH*-wild-type astrocytic tumors in the absence of promoter hypermethylation.

For *DIRAS-2* we detected significantly increased promoter methylation compared to non-neoplastic brain tissues in *IDH*-mutant astrocytic gliomas, but not in *IDH*-wild-type astrocytic gliomas and in *IDH*-mutant and 1p/19q-codeleted oligodendrogliomas. Altogether, methylation levels of the analyzed *DIRAS-2* CpG island were quite low, making it unlikely that promoter methylation alone accounts for the strong down-regulation of DIRAS-2 transcripts. Moreover, treatment of two glioblastoma cell lines with the demethylating agent 5’-aza-2’-deoxycytidine (AZA) did not consistently induce DIRAS-1 and -2 mRNA expression in both cell lines.

We then analyzed whether histone modifications, an important alternative mechanism of epigenetic gene silencing, may play a role in DIRAS-1 and DIRAS-2 transcriptional regulation. Two glioblastoma cell lines were treated with the histone deacetylase inhibitor trichostatin A (TSA), which increases histone acetylation and therefore leads to euchromatinization of transcriptionally active regions, such as promoter regions [29]. TSA treatment alone showed significant upregulation of DIRAS-1 expression in both cell lines and significant upregulation of DIRAS-2 in U251MG cells. DIRAS-2 expression was also increased in TSA-treated Hs683 cells; however, the results were not significant. Combinatory treatment with AZA and TSA led to significant increase of both DIRAS family members in both cell lines and even showed strong synergistic effects on DIRAS-1 and -2 expression in U251MG cells. Additional chromatin immunoprecipitation studies revealed a significant increase of the *DIRAS-1* and *DIRAS-2* promoter DNA bound to acetylated H3 in U251MG and Hs683 cells after TSA treatment. Chromatin immunoprecipitation analyses of glioblastoma and non-neoplastic brain tissue samples additionally confirmed the regulation of DIRAS-1 and -2 by histone modifications, as glioma tissues showed far less euchromatinization of the *DIRAS-1* and *-2* promoter regions. Taken together, these data suggest that the reduced expression of DIRAS-1 and DIRAS-2 in gliomas is driven by a combined mechanism of promoter hypermethylation and heterochromatinization.

Functionally, several studies showed that DIRAS-1 acts as a potential tumor suppressor by inhibiting cell proliferation and cell viability in different types of cancer, such as renal cell carcinoma, colorectal cancer, murine ovarian cancer cells, and esophageal squamous cell carcinoma, as well as gliomas [2,4,5,6] and that DIRAS-1 can also suppress tumor growth in nude mice [7]. However, we did not observe an impact of DIRAS-1 over-expression on cell proliferation in the two glioma cell lines analyzed. Our findings are in contrast to results reported by Ellis and colleagues, where DIRAS-1 over-expression inhibited cell proliferation [2]. This discrepancy could be due to the fact that we used a short-term 5-bromo-2’-deoxyuridine incorporation assay to measure newly synthesized DNA in replicating cells and Ellis and colleagues analyzed cell growth over 5 days by counting cell numbers at different time points. It might be possible that, for example, autophagy-induced cell death, which was reported in murine ovarian cancer cells [6], leads to reduced cell numbers after DIRAS-1 over-expression and not inhibition of cell proliferation. However, whether the mechanism of DIRAS-1-induced autophagic cancer cell death reported in ovarian cancer cells also applies to glioblastoma cells needs to be elucidated in additional analyses. 

Reports on the function of DIRAS-2 are quite sparse and also not consistent. Previous studies favor a tumor-suppressive function of DIRAS-2 by showing a role in autophagic cancer cell death, similar to DIRAS-1 in ovarian cancer [6], and that DIRAS-2 prevents the interaction of the noncanonical guanine nucleotide exchange factor SmgGDS with other pro-oncogenic, small GTPases, acting similarly to a dominant-negative small GTPase [10]. Another study in renal cell carcinoma, however, indicates a potential oncogenic function of DIRAS-2 because overexpression of DIRAS-2 in clear cell renal cell carcinoma cells led to increased cell proliferation, migration, and invasion in the absence of von Hippel–Lindau protein [11]. In our hands, over-expression of DIRAS-2 in glioblastoma cells did not significantly alter the proliferation of the glioblastoma cells and, therefore, our results cannot contribute to the discussion on whether DIRAS-2 in gliomas acts as tumor-suppressor or as oncogene. However, the fact that DIRAS-2 expression is commonly downregulated in gliomas argues in favor of a tumor suppressive role in these tumors.

Nevertheless, we can newly describe a role for DIRAS-1 and -2 in therapy-relevant processes. Standard treatment of glioma patients includes treatment with alkylating agents such as temozolomide or the combination of procarbazine, lomustine (CCNU), and vincristine (PCV) [30,31,32]. However, it is known that gliomas frequently develop resistance to these alkylating agents and, hence, recurrence of gliomas after chemotherapy is common [33,34,35,36]. Therefore, it is very important to gain additional insights into chemoresistance mechanisms to develop improved treatment strategies for glioma patients. Since there were reports on the role of DIRAS-3, another family member of small Ras-GTPases, in resistance to chemotherapeutic agents [20,37], we were interested to evaluate whether DIRAS-1 or -2 could mediate chemoresistance in glioblastoma cells. Indeed, overexpression of either DIRAS-1 or DIRAS-2 resulted in a significant sensitization of glioblastoma cells to the alkylating agent lomustine. Analyses on the protein level in glioblastoma cells showed that the altered sensitivity to lomustine might be regulated via Chk-2- and p53-dependent mechanisms. After lomustine treatment, we found increased p53 phosphorylation at serine 15 in DIRAS-1 and -2 transfected U251MG cells compared to control transfected cells. Phosphorylation at serine 15 can lead to reduced interaction of p53 with its negative regulator MDM2 that targets p53 for degradation by proteasomes. Moreover, phosphorylation of Ser15 is necessary for the apoptotic activity of p53 [38,39]. Possibly, DIRAS-1 or DIRAS-2 overexpression leads to increased p53-dependent apoptosis after DNA damage, rendering glioblastoma cells more susceptible to lomustine. We also observed increased Chk-2 phosphorylation at the threonine 68 residue after lomustine treatment in DIRAS-2 overexpressing Hs683 glioblastoma cells. Phosphorylation at threonine 68 is a prerequisite for a further activation of Chk-2 function [40,41,42]. Chk-2 can play multiple roles in DNA-damage repair: It can either be directly involved, it can activate cell cycle checkpoints, or it can induce p53-dependent apoptosis or senescence [41]. All these findings provide evidence for downregulation of DIRAS-1- and DIRAS-2 as a mechanism of chemoresistance in gliomas. The exact roles of DIRAS-1 and DIRAS-2 in activating p53- or Chk-2 dependent DNA-damage response, however, should be subject to further studies.

## 5. Conclusions

Our study establishes a relevance for DIRAS-1 and DIRAS-2 in gliomas. We found both genes downregulated by epigenetic mechanisms. Promoter methylation and histone modifications account for DIRAS1- and -2 inactivation. Our findings may be of predictive relevance as overexpression of DIRAS-1 and -2 was associated with a significantly higher sensitivity to the alkylating agent lomustin. The analysis of DNA-damage markers reinforces the notion that DIRAS-1 and -2 play a role in p53-dependent response to alkylating chemotherapy. 

## Data Availability

Prenormalized expression data sets from glioblastoma samples were obtained from the cancer genome atlas data portal (URL: https://tcga-data.nci.nih.gov, 2 December 2014) [13]. The glioblastoma sample data set published by Brennan et al. [13] was assessed for mutational analyses by using the cBioPortal webpage (https://www.cbioportal.org/; 22 June 2021) [15,16].

## Supplementary Materials

The following are available online at https://www.mdpi.com/article/10.3390/cancers13205113/s1, Table S1: DIRAS-1 and DIRAS-2 mRNA expression, CpG methylation status, and molecular characteristics of glioma tissues, non-neoplastic brain tissues, and a commercially available, universally methylated DNA (R132H: mutation in *IDH1*, substitution of the amino acid arginin to histidin in codon 132; R172K: mutation in *IDH*2, substitution of amino acid arginin to lysin in codon 172; LOH: loss of heterozygosity; RET: retention of heterozygosity for chromosome arms 1p and 19q, respectively). DIRAS-1 and DIRAS-2 mRNA expression and CpG island methylation scores in the glioblastoma cell lines U251MG and Hs683. Table S2: Primer sequences; Original and clearer figures in Figure S1; Original Western Blot images.

## References

[1] Kontani K., Tada M., Ogawa T., Okai T., Saito K., Araki Y., Katada T. (2002). Di-Ras, a Distinct Subgroup of Ras Family GTPases with Unique Biochemical Properties. J. Biol. Chem..

[2] Ellis C.A., Vos M.D., Howell H., Vallecorsa T., Fults D.W., Clark G.J. (2002). Rig is a novel Ras-related protein and potential neural tumor suppressor. Proc. Natl. Acad. Sci. USA.

[3] Bergom C., Hauser A.D., Rymaszewski A., Gonyo P., Prokop J.W., Jennings B.C., Lawton A.J., Frei A., Lorimer E.L., Aguilera-Barrantes I. (2016). The Tumor-suppressive Small GTPase DiRas1 Binds the Noncanonical Guanine Nucleotide Exchange Factor SmgGDS and Antagonizes SmgGDS Interactions with Oncogenic Small GTPases. J. Biol. Chem..

[4] Zheng R., Gao D., He T., Zhang M., Zhang X., Linghu E., Wei L., Guo M. (2017). Methylation of DIRAS1 promotes colorectal cancer progression and may serve as a marker for poor prognosis. Clin. Epigenetics.

[5] Xu X., Li J., Wang S., Zheng X., Xie L. (2018). RNAa and Vector-Mediated Overexpression of DIRAS1 Suppresses Tumor Growth and Migration in Renal Cell Carcinoma. Mol. Ther. Nucleic Acids.

[6] Sutton M.N., Yang H., Huang G.Y., Fu C., Pontikos M., Wang Y., Mao W., Pang L., Yang M., Liu J. (2018). RAS-related GTPases DIRAS1 and DIRAS2 induce autophagic cancer cell death and are required for autophagy in murine ovarian cancer cells. Autophagy.

[7] Zhu Y.-H., Fu L., Chen P.L., Qin Y.-R., Liu H., Xie F., Zeng T., Dong S.-S., Li J., Li Y. (2013). Downregulation of the Novel Tumor Suppressor DIRAS1 Predicts Poor Prognosis in Esophageal Squamous Cell Carcinoma. Cancer Res..

[8] Verma S.P., Agarwal A., Das P. (2018). Sodium butyrate induces cell death by autophagy and reactivates a tumor suppressor gene DIRAS1 in renal cell carcinoma cell line UOK146. Vitr. Cell. Dev. Biol. Anim..

[9] Reif A., Nguyen T.T., Weißflog L., Jacob C.P., Romanos M., Renner T.J., Buttenschøn H., Kittel-Schneider S., Gessner A., Weber H. (2011). DIRAS2 is Associated with Adult ADHD, Related Traits, and Co-Morbid Disorders. Neuropsychopharmacology.

[10] Ogita Y., Egami S., Ebihara A., Ueda N., Katada T., Kontani K. (2015). Di-Ras2 Protein Forms a Complex with SmgGDS Protein in Brain Cytosol in Order to Be in a Low Affinity State for Guanine Nucleotides. J. Biol. Chem..

[11] Rao H., Li X., Liu M., Liu J., Li X., Xu J., Li L., Gao W.-Q. (2020). Di-Ras2 promotes renal cell carcinoma formation by activating the mitogen-activated protein kinase pathway in the absence of von Hippel–Lindau protein. Oncogene.

[12] Louis D.N., Ohgaki H., Wiestler O.D., Cavenee W.K. (2016). WHO Classification of Tumours of the Central Nervous System.

[13] Brennan C.W., Verhaak R.G., McKenna A., Campos B., Noushmehr H., Salama S., Zheng S., Chakravarty D., Sanborn J.Z., Berman S.H. (2013). The Somatic Genomic Landscape of Glioblastoma. Cell.

[14] Schulze M., Violonchi C., Swoboda S., Welz T., Kerkhoff E., Hoja S., Brüggemann S., Simbürger J., Reinders J., Riemenschneider M.J. (2017). RELN signaling modulates glioblastoma growth and substrate-dependent migration. Brain Pathol..

[15] Gao J., Aksoy B.A., Dogrusoz U., Dresdner G., Gross B., Sumer S.O., Sun Y., Jacobsen A., Sinha R., Larsson E. (2013). Integrative Analysis of Complex Cancer Genomics and Clinical Profiles Using the cBioPortal. Sci. Signal..

[16] Cerami E., Gao J., Dogrusoz U., Gross B.E., Sumer S.O., Aksoy B.A., Jacobsen A., Byrne C.J., Heuer M.L., Larsson E. (2012). The cBio Cancer Genomics Portal: An Open Platform for Exploring Multidimensional Cancer Genomics Data. Cancer Discov..

[17] Dietmaier W., Lorenz J., Riemenschneider M. (2015). Molekulare Diagnostik in der Neuropathologie. Der Pathol..

[18] Jiang Y., Matevossian A., Huang H.-S., Straubhaar J., Akbarian S. (2008). Isolation of neuronal chromatin from brain tissue. BMC Neurosci..

[19] Schmidt N., Windmann S., Reifenberger G., Riemenschneider M.J. (2011). DNA Hypermethylation and Histone Modifications Downregulate the Candidate Tumor Suppressor Gene RRP22 on 22q12 in Human Gliomas. Brain Pathol..

[20] Washington M.N., Suh G.S.B., Orozco A.F., Sutton M., Yang H., Wang Y., Mao W., Millward S.W., Ornelas A., Atkinson N.S. (2015). ARHI (DIRAS3)-mediated autophagy-associated cell death enhances chemosensitivity to cisplatin in ovarian cancer cell lines and xenografts. Cell Death Dis..

[21] Riemenschneider M.J., Reifenberger J., Reifenberger G. (2008). Frequent biallelic inactivation and transcriptional silencing of theDIRAS3 gene at 1p31 in oligodendroglial tumors with 1p loss. Int. J. Cancer.

[22] Peng Y., Jia J., Jiang Z., Huang D., Jiang Y., Li Y. (2018). Oncogenic DIRAS3 promotes malignant phenotypes of glioma by activating EGFR-AKT signaling. Biochem. Biophys. Res. Commun..

[23] Zhong C., Shu M., Ye J., Wang X., Chen X., Liu Z., Zhao W., Zhao B., Zheng Z., Yin Z. (2019). Oncogenic Ras is downregulated by ARHI and induces autophagy by Ras/AKT/mTOR pathway in glioblastoma. BMC Cancer.

[24] Chen J., Shi S., Yang W., Chen C. (2014). Over-expression of ARHI decreases tumor growth, migration, and invasion in human glioma. Med Oncol..

[25] Zou C.-F., Jia L., Jin H., Yao M., Zhao N., Huan J., Lu Z., Bast R.C., Feng Y., Yu Y. (2011). Re-expression of ARHI (DIRAS3) induces autophagy in breast cancer cells and enhances the inhibitory effect of paclitaxel. BMC Cancer.

[26] Mao Y., Han Y., Shi W. (2017). The expression of *aplysia ras homolog I* (*ARHI*) and its inhibitory effect on cell biological behavior in esophageal squamous cell carcinoma. OncoTargets Ther..

[27] Tang H.-L., Hu Y.-Q., Qin X.-P., Jazag A., Yang H., Yang Y.-X., Yang X.-N., Liu J.-J., Chen J.-M., Guleng B. (2012). Aplasia ras homolog member I is downregulated in gastric cancer and silencing its expression promotes cell growth in vitro. J. Gastroenterol. Hepatol..

[28] Turcan S., Rohle D., Goenka A., Walsh L., Fang F., Yilmaz E., Campos C., Fabius A.W.M., Lu C., Ward P. (2012). IDH1 mutation is sufficient to establish the glioma hypermethylator phenotype. Nature.

[29] Schones D.E., Zhao K. (2008). Genome-wide approaches to studying chromatin modifications. Nat. Rev. Genet..

[30] Wick W., Hartmann C., Engel C., Stoffels M., Felsberg J., Stockhammer F., Sabel M.C., Koeppen S., Ketter R., Meyermann R. (2009). NOA-04 Randomized Phase III Trial of Sequential Radiochemotherapy of Anaplastic Glioma With Procarbazine, Lomustine, and Vincristine or Temozolomide. J. Clin. Oncol..

[31] Reifenberger G., Wirsching H.-G., Knobbe-Thomsen G.R.C.B., Weller M. (2016). Advances in the molecular genetics of gliomas—implications for classification and therapy. Nat. Rev. Clin. Oncol..

[32] Stupp R., Mason W.P., Bent M.V.D., Weller M., Fisher B., Taphoorn M.J., Belanger K., Brandes A., Marosi C., Bogdahn U. (2005). Radiotherapy plus Concomitant and Adjuvant Temozolomide for Glioblastoma. New Engl. J. Med..

[33] Weil S., Osswald M., Solecki G., Grosch J., Jung E., Lemke D., Ratliff M., Hänggi D., Wick W., Winkler F. (2017). Tumor microtubes convey resistance to surgical lesions and chemotherapy in gliomas. Neuro-Oncology.

[34] He H., Yao M., Zhang W., Tao B., Liu F., Li S., Dong Y., Zhang C., Meng Y., Li Y. (2015). MEK2 is a prognostic marker and potential chemo-sensitizing target for glioma patients undergoing temozolomide treatment. Cell. Mol. Immunol..

[35] Beier D., Schulz J.B., Beier C.P. (2011). Chemoresistance of glioblastoma cancer stem cells-much more complex than expected. Mol. Cancer.

[36] Happold C., Roth P., Wick W., Schmidt N., Florea A.-M., Silginer M., Reifenberger G., Weller M. (2012). Distinct molecular mechanisms of acquired resistance to temozolomide in glioblastoma cells. J. Neurochem..

[37] Xiang S., Dauchy R.T., Hoffman A.E., Pointer D., Frasch T., Blask D.E., Hill S.M. (2019). Epigenetic inhibition of the tumor suppressor ARHI by light at night-induced circadian melatonin disruption mediates STAT3-driven paclitaxel resistance in breast cancer. J. Pineal Res..

[38] Chehab N.H., Malikzay A., Stavridi E.S., Halazonetis T.D. (1999). Phosphorylation of Ser-20 mediates stabilization of human p53 in response to DNA damage. Proc. Natl. Acad. Sci. USA.

[39] Shieh S.-Y., Ikeda M., Taya Y., Prives C. (1997). DNA Damage-Induced Phosphorylation of p53 Alleviates Inhibition by MDM2. Cell.

[40] Matsuoka S., Rotman G., Ogawa A., Shiloh Y., Tamai K., Elledge S.J. (2000). Ataxia telangiectasia-mutated phosphorylates Chk2 in vivo and in vitro. Proc. Natl. Acad. Sci. USA.

[41] Zannini L., Delia D., Buscemi G. (2014). CHK2 kinase in the DNA damage response and beyond. J. Mol. Cell Biol..

[42] Melchionna R., Chen X.-B., Blasina A., McGowan C.H. (2000). Threonine 68 is required for radiation-induced phosphorylation and activation of Cds1. Nature.

